# A randomized controlled trial investigating the effects of PCSO-524®, a patented oil extract of the New Zealand green lipped mussel (*Perna canaliculus*), on the behaviour, mood, cognition and neurophysiology of children and adolescents (aged 6–14 years) experiencing clinical and sub-clinical levels of hyperactivity and inattention: study protocol ACTRN12610000978066

**DOI:** 10.1186/1475-2891-12-100

**Published:** 2013-07-16

**Authors:** James D Kean, David Camfield, Jerome Sarris, Marni Kras, Richard Silberstein, Andrew Scholey, Con Stough

**Affiliations:** 1 Centre for Human Psychopharmacology, Swinburne University of Technology, Melbourne, Australia; 2 Department of Psychiatry, University of Melbourne, Melbourne, Australia

**Keywords:** Lyprinol, Omega XL, ADHD, Hyperactivity, Impulsivity, Omega 3 s, Marine oil extract, RCT

## Abstract

**Background:**

The prevalence rate of attention-deficit/hyperactivity disorder (ADHD) within Western cultures is between 5% and 12%, and is the most common psychiatric illness among school-aged children, with an estimated 50% of these children retaining ADHD symptoms for the rest of their lives. Children with ADHD have lower blood levels of long-chain Poly Unsaturated Fatty Acids (LC PUFAs) compared with children without ADHD, and following PUFA supplementation, have shown improvements in ADHD-related symptoms. One highly promising marine based LC PUFA preparation is the Omega-3-rich Lyprinol/Omega XL which is a natural formulation containing standardised lipid extract of the New Zealand green lipped mussel (*Perna canaliculus*) known as PCSO-524® which contains a unique combination of free fatty acids, sterol esters, polar lipids and carotenoids. It is this unique combination of marine lipids that may assist in correcting the decreased levels of LC PUFA levels in children with symptoms of ADHD. The compound is a mixture belonging to a lipid group called sterol esters (SE). The fatty acids in the SE fraction are mainly myristic acid, palmitic acid, palmitoleic acid, stearic acid, oleic acid, linoleic acid, eicosapentaenoic acid (EPA), and docosahexaenoic acid (DHA). Lyprinol/Omega XL has previously been shown to contain a potent group of Omega-3 lipids that block the 5 - lipoxygenase metabolic pathway responsible for inflammation in the body.

**Methods:**

A randomized double blind placebo controlled trial will be utilized to assess the effects of 14 weeks administration of Lyprinol/Omega XL versus placebo in 150 children aged 6 to 14 years with high levels of hyperactivity and inattention. Additionally, a range of cognitive, mood and central electrophysiological measures will be undertaken during the 14 week supplementation trial. The primary outcome measure, the Conners’ Parent Rating Scales will be completed initially at baseline, then in weeks 4, 8, 10, 14 and then again at 4 weeks post-administration (week 18). The results will contribute to our understanding of the efficacy of marine based Omega-3 s with high anti-inflammatory actions on inattention and hyperactivity in children aged 6 to 14 years.

## Background and rationale

Developmental disorders can have detrimental effects on a child’s social, emotional and academic future. Attention-deficit/hyperactivity disorder (ADHD) is the most prevalent developmental disorder in school aged children [1-3]. The symptoms of ADHD include hyperactivity, impulsivity, and inattention and are common traits in other similar developmental disorders at both clinical and sub-clinical levels [4-6]. This subclinical group highlights an area of child mental health that is wide-spread and largely overlooked. A child’s ability to attend, recall and utilise learnt information inside and outside of the classroom is vulnerable to a number of environmental influences and genetic predispositions. Links to dysfunctions of neuro-circuitry [7,8], neuro-chemistry [9], developmental deviations [10,11] and neuronal maturation [12] have all been investigated as suspected causes for ADHD. One treatment model is based on the reduced levels of long chain poly unsaturated fatty acids (LC PUFAs) in the plasma of ADHD children compared to normals [6,13-15]. This has led to extensive research examining the efficacy of omega-3 supplementation as a natural alternative to methylphenidate (MPH e.g., Ritalin®) and other stimulant and non-stimulant pharmaceuticals [16,17].

### What is attention-deficit/hyperactivity disorder?

The prevalence rate of ADHD within Western cultures is between 5% and 12%, representing the most common psychiatric illness among school-aged children, with an estimated 50% of these children retaining ADHD symptoms into adulthood and for the rest of their lives [1,3] and less than 5% of this demographic attaining a university degree [18]. The disorder involves a child’s inability to maintain attention and focus and to remain calm, quiet and co-operative in both school and home settings. Symptoms of the disorder include inattention and/or excessive hyperactivity and impulsivity. A clinical diagnosis of the disorder involves one of three forms: *Predominantly Inattentive Type* (mainly symptoms of inattention), *Hyperactive-Impulsive Type* (mainly excessive behaviour related symptoms) or *Combined Type* (symptoms of both inattention and hyperactivity/impulsivity are clearly evident) [2].

Neurological causes of the disorder have been linked to dysfunctions of the Prefrontal Cortex (PFC) [7,8]. The PFC controls and moderates cognitive processes including executive functioning which includes decision making, planning and monitoring behaviours. Theories on the dysfunction of the neuro-circuitry within the prefrontal cortex have provided some insight into potential causes and are based on two developmental complications; maturational lag [12], or developmental deviation [10,11]. Depending on the severity of symptoms of the child, it has also been argued that this maturational lag in most children will gradually be reduced relative to healthy normal peers [19]. Developmental deviation has been found in electroencephalograph (EEG) studies revealing that despite age changes, ADHD symptoms and associated cognitive differences remain as maturation continues, suggesting an abnormal developmental path to peers [10,11]. The most common theories describing neurochemical causes of the disorder centre on low amounts of the catecholamine neurotransmitters dopamine and noradrenaline in the PFC [20,21]. There has been extensive research into these causes including genetic influences that predispose a child to deficits in dopamine (and serotonin) transmission [22]. Other causes have been attributed to exposure of the foetus/child to harmful agents in the prenatal, perinatal, postnatal and early childhood phases [23]. *In utero* exposure to excess alcohol, tobacco and lead have been linked to an increased risk of ADHD [4], while studies on diet have found that ADHD symptoms may become exacerbated when certain additives or food preservatives are consumed [24].

Stimulant medication such as methylphenidate (MPH e.g., Ritalin®), are considered to be beneficial for ADHD children as it is thought that administration of MPH increases levels of noradrenalin and dopamine in the PFC [1]. Animal models have demonstrated the efficacy of MPH by highlighting a specific mode of action of MPH in the dorsomedial PFC and linking this to cognitive performance in male Sprague–Dawley rats [25]. Some studies have also demonstrated effectiveness of MPH in treating those children with intellectual disabilities (IQ 30–69) and these disabilities have been known to increase the severity of ADHD symptomology [26]. Problems with the chronic use of these medications however, can lead to complications with appetite, trouble sleeping and increased levels of anxiety and a host of other unpleasant side effects [26-28]. Therefore it is not uncommon for parents to seek alternative treatments that do not have the same side effect profile as MPH for their children [29-31].

### The omega-3 model

Observations by Dyerberg and Bang (1986) found that the Eskimos of Greenland had low incidence rates of coronary heart disease due to their high fat diets dominated by the LC PUFA omega-3 [32]. This finding lead to further research into the actions of omega-3 s on cognitive, behavioural and cardiovascular functions. Children with ADHD have presented with lower levels of LC PUFAs in the blood compared with normal populations and following supplementation, have shown improvements in symptoms related to ADHD [15,17]. There are several reasons why these children may have insufficient LC PUFAs such as: low levels of the LC PUFAs obtained in the diet; the inefficient conversion of shorter chain PUFAs (SC PUFAs) into LC PUFAs; or the increased speed of metabolism of LC PUFAs [33]. Omega-3 s increase the malleability of neuronal cells, allowing more efficient nutrient exchange, increased cellular nourishment while also increasing fluidity of the blood [34,35]. Increased membrane fluidity is associated with faster neural transmission and more efficient biochemical performance [34]. Fontani et al. (2005) discovered that Omega-3 supplementation significantly improved higher brain functions including sense of well-being, reactivity, attention, cognitive performance, and mood in young healthy adults [36].

The New Zealand Green Lipped Mussel (*Perna canaliculus*) dates back to prehistoric times with coastal Maori folklore claiming that consumption of the mussels aided better health and fewer cases of arthritis than New Zealanders residing further inland [37]. In a trial designed in the 1960s to find cancer fighting therapies derived from various marine organisms, researchers found patients reported relief from symptoms of arthritis following the administration of the powdered form of *P. canaliculus*[38]. Since this time, research on *P. canaliculus* has shown benefits to cardiovascular functioning and anti-inflammatory processes [39-41]. Rheumatic disorders are second only to heart disease in the ageing population, making them one of the most prevalent and costly disorders in adults stimulating the increased research into *P. canaliculus*[42]. With inconsistent research results using non-steroidal anti-inflammatories (NSAIDs) [43] there has been significant use of natural medicines for rheumatic disorders [44]. The process of extracting the marine lipids from the *P. canaliculus* involves introducing tartaric acid to the freeze dried powder form under a patented mussel stabilization process [45]. This method was introduced following the inconsistent anti-inflammatory effects of *P. canaliculus* from batch to batch [46]. A recent systematic review on the freeze-dried powdered form of the *P. canaliculus* showed mixed evidence for its effects on osteoarthritis and rheumatoid arthritis, despite the preservation of its marine lipids [42].

### Lyprinol®/omega XL (PCSO-524®)

Lyprinol®/Omega XL is a unique oil-based form of *P. canaliculus.* A patented liquid carbon dioxide super critical fluid extraction process (PCSO-524®) is unique to Lyprinol®/Omega XL creating a highly condensed form of the marine lipids of *P. canaliculus*[47]. Studies on PCSO-524® have shown that this extract promotes anti-leukotriene production, which can reduce symptoms of asthma in a study population of young children [40]. It was this research that led to the confirmation of the safety and tolerability of PCSO-524® in a population of children as well as previous research demonstrating its efficacy and safety in adults [41,48,49]. The unique grouping of fatty acids in PCSO-524® has evolved as the most potent group of Omega-3 lipids in blocking the 5 - lipoxygenase metabolic pathway responsible for inflammation in the body [50]. It is this potent level of marine lipids that may assist in the decreased PUFA levels in children with symptoms of ADHD and similar developmental disorders and which is the major (but not only) mechanistic basis of the current clinical trial.

### Design

The study is a randomized, double-blind, placebo-controlled, 2-armed, parallel groups clinical trial with children and adolescents randomized to receive either 3 capsules (if less than or equal to 45 kg) or 4 capsules (if greater than 45 kg) of PCSO-524® or placebo.

### Study aims and hypotheses

The objective of this trial is to examine whether 14 week administration of a naturally occurring combination of omega 3′s improves a range of cognitive (measured with the COMPASS Cognitive Test Battery), mood (Brunel Mood Scales), behavioural (Conners’ Parent Rating Scale) and psychophysiological (electroencephalography and steady state topography) measures in children aged 6–14 years with symptoms of inattention and hyperactivity relative to placebo. It is anticipated that this project will conclude all testing in late 2013.

The primary aim of the current study is to examine the levels of hyperactivity, impulsivity and inattention in children aged 6 to 14 years of age before, during and after receiving a 14-week intervention of Lyprinol® compared to placebo. The primary outcome will be examining parental reports using the *Conners’ Parent Rating Scales −3* approximately every 4 weeks [51,52]. The Conners’ Parent Rating Scales (CPRS) were developed as a comprehensive checklist for acquiring parental reports of the basic presenting problems [52]. Secondary outcomes will also include the acquisition of resting state electroencephalography (EEG) in two states of eyes open and eyes closed. Changes in brain wave ratios in key areas of activity (prefrontal cortex) from baseline, week 8 and week 14 will also be examined to test the hypothesis that Lyprinol® will ameliorate the cortical hypoarousal that has been associated with ADHD [53] and increase the ratio of *alpha/beta* waves compared to *placebo*. Cognitive changes will be monitored using the Test of Variables of Attention (TOVA) as well as the Computerised Mental Performance Assessment System (COMPASS) [54]. Mood will be examined closely using the Brunel Mood Scale (BRUMs) for adolescents [55].

### Centres

All cognitive, mood and electrophysiological testing will take place within the Swinburne Centre for Human Psychopharmacology at Swinburne University in Victoria Australia. Additional CPRS will be issued to the parents of the participants who will be completing these questionnaires in the home environment in weeks 4, 10 and 18 (4 weeks post-administration of either Lyprinol or placebo).

### Participants

One hundred and fifty children aged between 6 and 14 years will complete either 14 week consumption of Lyprinol/Omega XL or placebo. This age range is one of the most common developmental stages for children to experience increased levels of hyperactivity, impulsivity and inattention and where 5-12% of children will receive a diagnosis of ADHD. The study was ethically approved by the Swinburne University Human Research Ethics Committee (SUHREC Project 2010/175) and all participants will provide written informed consent. All procedures will be conducted in accordance with the Declaration of Helsinki (2008) and the good clinical practice (GCP) guidelines. The trial has been registered with the Australian and New Zealand Clinical Trials Registry (ACTRN12610000978066).

### Inclusion and exclusion criteria

#### Inclusion criteria

Healthy, non-smoking males and females aged between 6 and 14 years; DSM-IV ADHD rating score above 15; Fluent in English; Parent/legal guardian to provide a personally signed and dated informed consent indicating that they have been informed of all pertinent aspects of the trial; the parent will then get verbal consent from the child.

#### Exclusion criteria

Medical diagnosis other than ADHD, Oppositional defiant disorder or similar behavioural disorders; Currently taking any medication (other than stimulants if a formal diagnosis of ADHD or other behavioural disorder is present); Current or history of heart disease or high blood pressure or diabetes; Health conditions that would affect food metabolism including the following: food allergies, kidney disease, liver disease and/or gastrointestinal diseases (e.g. Irritable bowel syndrome, coeliac disease, peptic ulcers); Pregnant or breast feeding; Unable to participate in all scheduled visits, treatment plan, tests and other trial procedures according to the protocol; Allergy to shellfish; Epilepsy or photosensitive.

### Treatments

The trial treatments are a naturally occurring combination of omega-3 s (Lyprinol®/Omega XL), and placebo. Participants must take either 3 × or 4 × 260 mg capsules in the morning (with breakfast). *Principal Ingredients per 260 mg capsule for the tablets are:* Natural mono-unsaturated Olive oil - 100 mg; PCSO-524® GLM pat. lipids (Eicosatetraenoic acid) - 50 mg; and Vitamin E (D-alpha-Tocepherol) as antioxidant - 0.225 mg. The Lyprinol capsules contain 18% EPA (46.8 mg) and 14% DHA (36.4 mg) per capsule. The placebo capsule contains 35.5 mg of olive oil, 112 mg of lecithin, 12 mg of coconut oil and 0.5 mg of 30% beta-carotene. Both treatments are contained within tablets consisting of: Gelatin; Sorbitol syrup; Glycerin. The placebo capsule matches the Lyprinol® capsule in touch, taste, smell and size.

In terms of the PCSO-524® component in each Lyprinol/Omega XL capsule: Lyprinol® is a lipid extract of the New Zealand green lipped mussel (*Perna canaliculus*). Its major active constituents are unsaturated fatty acids. The amount of these fatty acids ranges between 6.3% and 9.5% for the omega-6 class, and between 9.8% and 12.15% for the omega-3 class. Lyprinol/Omega XL is currently listed with the TGA of Australia as an anti-inflammatory. Lyprinol/Omega XL has been shown to be a reproducible, relatively stable, source of bioactive lipids with a much greater potency than plant/marine oils currently used as nutritional supplements to ameliorate signs of inflammation. Experimental studies have shown that lipid extracts are effective at modulating 5’lipoxygenase and cyclo-oxygenase pathways, which are responsible for the production of eiconoids, including leukotrienes and prostaglandins. The lipid extract of the New Zealand green lipped mussel (*Perna canaliculus*), marketed under the brand name Lyprinol and Omega XL, is rich in eicosapentaenoic acid (EPA) and docosahexanoic acid (DHA), omega-3 fatty acids that inhibit the metabolism of arachidonic acid. The compound is a mixture belonging to a lipid group called sterol esters (SE). The fatty acids in the SE fraction are mainly myristic acid, palmitic acid, palmitoleic acid, stearic acid, oleic acid, linoleic acid, EPA and DHA. The sterols found in this fraction included cholesterol, cholesta-3, 5-diene, 26, 27-dinoergostadienol, cholesta-5, 22-dien-3-ol, ergosta-5, 22-dien-3-ol.

### Procedure

Participants will be randomly allocated to a coded treatment group and they will be given a supply of their treatment. Participants will be required to take either 3 or 4 capsules daily (in the morning with breakfast) for the following 14 weeks. The schedule for testing is presented in Table 1. Weight plays a large role in the digestion and absorption of nutrients giving reason for the researcher’s choice of a weight limit (± 45 kg) over that of an age limit in order to determine how many capsules each child would take.

**Table 1:** Behavioural and demographic measures

	**V1**	**V2**		**V3**		**V4**	
	**Week 1**	**Week 2**	**Week 4**	**Week 8**	**Week 10**	**Week 14**	**Week 18**
	**Practice**	**Centre**	**Home**	**Centre**	**Home**	**Centre**	**Home**
Structured Interview	X						
(DSM ADHD Rating)							
Conners’ Parent Rating Scale		X	X	X	X	X	X
Global Clinical Impressions Scale		X		X		X	
Current Health & Medical Questionnaire	X	X	X	X	X	X	
Demographics Questionnaire	X						
Omega-3 Intake/Food Diary	X			X			
***Cognitive and Psychophysiological Measures***							
COMPASS cognitive battery	X	X		X		X	
Test of Variables of Attention (TOVA)	X	X		X		X	
Brunel Mood Scale (BRUMS)	X	X	X	X	X	X	X
EEG Resting State		X		X		X	
Steady State Topography (SST)		X		X		X	

The first centre visit is a practice day where participants complete screening questionnaires and familiarise themselves with the study procedures and tests. The next Centre visit is a baseline session where participants complete all tests (including the EEG) and are randomly allocated to receive one of the two treatments (Lyprinol®/placebo) for the next 14 weeks. Testing for all subsequent centre visits will follow the same outline of their baseline session with follow up visits at weeks 8 and 14. In between these times, in weeks 4 and 10, the parents will complete a CPRS and health questionnaire while the participants will complete a mood questionnaire used in previous visits. As the study progresses researchers will contact the participants to determine compliance rates for the daily use of the intervention as well as any adverse events. Throughout the duration of the study, parents will be required to complete the CPRS at home and bring in the completed questionnaire each session or at the end of the study. The participants will be given a treatment compliance diary and participants will need to mark each day they take their treatment (this is to monitor for compliance). At the end of the study, participants will be required to return the compliance diary and symptom checklists to the researcher as well as any remaining treatment. The parent will also complete a seven-day food diary in order to determine if the child’s omega-3 intake from non-trial sources has remained consistent since visit 1.

### Post-treatment period: follow up

**Figure 1:**
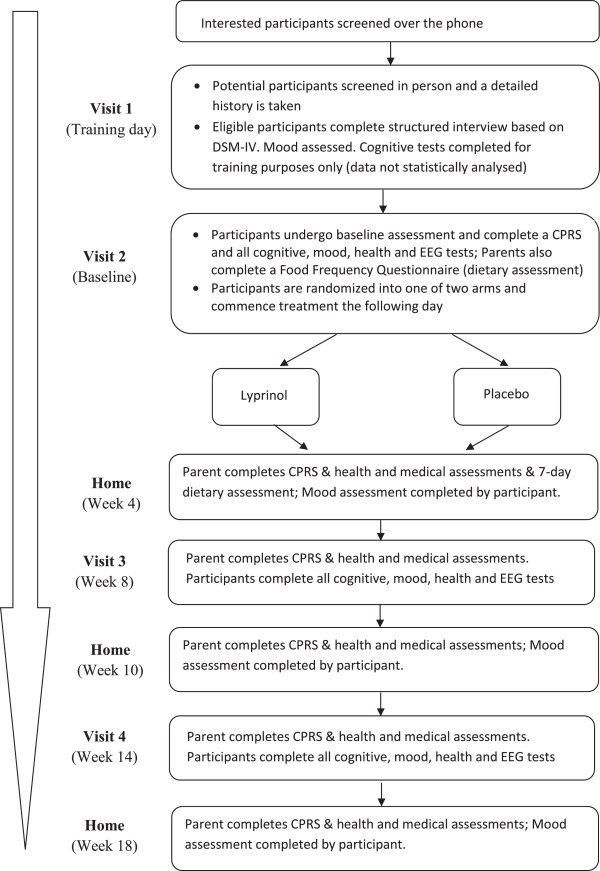
Lyprinol protocol flow diagram.

Participants’ parents will complete the CPRS and the Brunel Mood Scale four weeks following the cessation of Active or placebo administration to determine any changes in mood or behaviour since ceasing treatment. These scales can be completed by the parents independently or (more usually) with the child. Participants will be questioned non-specifically for any Adverse Events (AEs). A review of their Symptom Checklists will also help to determine if a participant experienced an AE. Figure 1 describes the flow of data collection for this study.

#### Randomization and safety

Randomization of participants to treatment groups will be determined by random allocation. All 150 participants will be assigned to treatment group A or B using a computer generated random number generator by a neutral third party. Eligible, recruited participants will be assigned a participant number. The treatment number that has been placed next to the participant’s number will be the allocated treatment for that individual. Blinding will be achieved by enlisting a person outside of the project to code the treatments, and maintain the key to this code until data collection is completed. An emergency code break envelope will be provided to the principle investigator which will only be opened in case of emergency. Participants’ parents will also be given a weekly symptom checklist to complete to monitor for any adverse events. Researchers will be blinded to the treatment conditions.

#### Primary outcome

The CPRS will be used to record parents’ ratings of inattention and hyperactivity. The CPRS is to be filled out by the parent at home on weeks 2, 4, 8, 10, 14 and 18 [56]. Researchers involved exclusively in this trial will score and enter the data from each CPRS following the participants’ completion of the trial. The researchers are blinded to treatment allocation.

#### Secondary outcome measures

The Tests of Variables of Attention (TOVA) will assess symptoms of inattention and impulsivity. The TOVA is a computer-based assessment of inattention [54].

### COMPASS (computerised mental performance assessment system)

The COMPASS battery has been developed to include tests which have been shown to be sensitive to nutritional manipulations. The tasks in this study are designed to allow assessment across the major cognitive domains i.e. attention, working memory, secondary memory and executive function. Parallel versions of each of the following tasks allowed for multiple testing. Previous studies have used a similar customized battery in testing adolescents and their responses to dietary changes [57-63]. The following cognitive COMPASS tasks will be administered: Word presentation; Immediate word recall; Picture presentation; Simple reaction time; Choice reaction time; Numeric working memory; Delayed word recall; Delayed word recognition; Delayed picture recognition (see Table 2 for task descriptions).

**Table 2:** COMPASS task descriptions and scoring

**Task**	**Task description**	**Task scoring**
Word presentation	Individual words are presented sequentially on the monitor. Stimulus duration was 1 s, as is the inter-stimulus interval.	
Immediate word recall	The participant is allowed 60 s to write down as many of the words as possible.	The task is scored for number of correct answers, errors and intrusions and the resulting score is converted into a percentage
Picture presentation	Twenty line-drawings of everyday items are presented on the computer screen. Stimulus duration is 3 s, with a 1 s inter-stimulus duration.	
Simple reaction time	A series of upwards pointing arrows appear in the centre of the screen with a randomly varying inter-stimulus interval between 1 and 3 s. The participant will press the space bar as quickly as possible to the on screen appearance of each stimulus.	The task is scored in Response times (recorded in milliseconds)
Choice reaction time	Arrows pointing to the left or right are presented in the centre of the screen with a randomly varying inter-stimulus interval of 1–3 s. The volunteer presses the corresponding ‘left’ or ‘right’ cursor key on the computer keyboard as quickly as possible.	The task is scored for accuracy (%) and reaction time (ms).
Numeric working memory	A series of five digits is presented on the computer screen sequentially for the participants to hold in their memory. This is followed by a series of 30 probe digits. The participants indicate whether or not the digit was from the original series by pressing corresponding keys labelled ‘yes’ and ‘no’. This is repeated three further times with different stimulus sets.	Reaction times (ms) and accuracy (% correct) are measured
Delayed word recall	Approximately 20 min after the word presentation, the participant is allowed 60 s to write down as many of the items from word presentation as possible.	The task is scored as for immediate word recall.
Delayed word recognition	Word recognition is tested by representation of the words from the original list randomly interspersed with an equal number of distracter words. Participants respond either ‘yes’ or ‘no’ by pressing corresponding key to indicate whether the word had previously been presented or not.	The task is scored for accuracy (%), and reaction time (ms).
Delayed picture recognition	Picture recognition is tested by the presentation of the original drawings and an equal number of distracters in random order. Participants respond either ‘yes’ or ‘no’ by key press in order to indicate whether the picture had been presented previously.	The task is scored for accuracy (%) and reaction time (ms).

### Mood

The Brunel Mood Scale (BRUMS) was derived from the Profile of Mood States mood scale [64] and contains 24 simple mood descriptors such as angry, energetic, nervous, and unhappy. The BRUMS (formerly known as the profile of Mood States – Adolescents or POMS-A) was designed for purely adolescent populations and the validation process can be found in Terry et al [55,65].

### Diet

At the beginning of the trial the parent will complete a Food Frequency Questionnaire (FFQ) that will provide a detailed description of the child’s food intake. In week 4 of the trial the parent will complete a 7-day food diary consisting of what the child has had to eat for breakfast, lunch and dinner as well as any significant snacks throughout those days. This diary will be compared to the FFQ to determine any changes in diet that may have occurred while in the study.

### Resting electroencephalography (EEG) and steady state topography (SST)

Brain electrical activity will be recorded from 64 sites on the scalp, including all of the International 10–20 positions and sites located midway between the 10–20 locations. All electrodes will be held in place using an electrocap. Electrode impedance will vary from 2.5 kohmsto 8.5 kohms when measured at 40 Hz. The average of both earlobes will serve as a reference. EEG will be amplified and bandpass filtered (3 dB down at 0.1 Hz and 30 Hz) prior to digitisation to 12-bit accuracy at a rate of 200 Hz. Changes in brain wave ratios in key areas of activity will also be examined to test the hypothesis that Lyprinol® will ameliorate the cortical hypoarousal that has been associated with ADHD [11,53,66-71]. The stimulus used to evoke the SST will be a diffuse 13 Hz sinusoidal flicker superimposed on the visual field by a pair of goggles. The goggles will comprise two half-silvered mirrors that reflect two light-emitting diode arrays over the subject’s visual field, subtending a horizontal angle of 160 degrees and a vertical angle of 90 degrees. The maximum intensity at the peak of the stimulus waveform will be 5.0 cd/m^2^ and the modulation depth will be 45%. In order to control for variations in vigilance, subjects will perform a low level cognitive task.

#### Signal processing of EEG data

Two hundred and fifty seconds of EEG data will be gathered for each subject in each condition, yielding a frequency resolution of 0.004 Hz. The SST recorded at each electrode will be determined via Fourier analysis centred at 13 Hz with a width of 0.004 Hz. The sine and cosine Fourier components will be used to determine the amplitude and phase of the SST [72]. Data will be checked for artefact using techniques described in Silberstein et al., (1995) [73].

### Global clinical impression scale (GCI)

Amongst the most widely used of extant brief assessment tools in psychiatry, the GCI is a 3-item observer-rated scale that measures illness severity (CGIS), global improvement or change (GCIC) and therapeutic response. The GCI has proved to be a robust measure of efficacy in many clinical drug trials, and is easy and quick to administer. The GCI is rated on a 7-point scale, with the severity of illness scale using a range of responses from 1 (normal) through to 7 (amongst the most severely ill patients). GCIC scores range from 1 (very much improved) through to 7 (very much worse). Treatment response ratings should take account of both therapeutic efficacy and treatment-related adverse events and range from 0 (marked improvement and no side-effects) and 4 (unchanged or worse and side-effects outweigh the therapeutic effects). Each component of the GCI is rated separately; the instrument does not yield a global score.

#### Power and statistical analysis

The sample size for this study is 150 participants with 75 participants in each arm (Lyprinol vs Placebo). A power calculation to determine the sample size was performed using Gpower 3.12. Based on previous studies looking at omega-3 supplementation in children with ADHD such as Sinn & Bryan (2007) who had an *n* of 132 and were able to find statistical significance in terms of the CPRS [15], a small to medium effect size (F = .20) on the Conners’ was postulated for Lyprinol over placebo from baseline to week 18 over six time points (critical F = 3.92, with an alpha probability of 0.05 and beta power of 0.80). This provided a sample size of 118; with drop-outs being projects at approximately 20%, a sample of 150 participants was estimated. Repeated Measures ANOVAs with an *n* of 150 (75 per group) will allow the researchers to account for any confounding variables in the trial including within-group and between-group variability. Any heterogeneous variables showing statistically significant differences at baseline will be co-varied in order to negate their influence on the power of the study.

## Conclusions

Attention-deficit/hyperactivity disorder (ADHD) is the most prevalent developmental disorder in school aged children [1,3]. The symptoms of ADHD include hyperactivity, impulsivity, and inattention. The prevalence rate of ADHD within Western cultures is between 5% and 12%, representing the most common neurodevelopmental disorder among school-aged children, with an estimated 50% of these children retaining ADHD symptoms into adulthood and for the rest of their lives [1,3] and less than 5% of this demographic attaining a university degree [18]. Interventions which are efficacious and which have no or little side effects are urgently required for both children and adolescents who fall within the clinical classification of ADHD and also who have high levels of the symptoms of inattention and impulsivity but who fall below the classification of ADHD. One treatment model is based on the reduced levels of LC-PUFAs in the plasma of ADHD children compared to normals [6,13-15,17]. This has led to extensive research examining the efficacy of omega-3 supplementation as a natural alternative to methylphenidate (MPH e.g., Ritalin®) and other stimulant and non-stimulant pharmaceuticals [16,17]. In the current study we will administer either placebo or a novel and patented marine lipid from the New Zealand Green Lip Mussel (PCSO-524®) to 150 children and adolescents with ADHD or high levels of inattention and hyperactivity for 14 weeks. A range of behavioural and neurophysiological measures will be undertaken. The results of the study will aid our understanding of the role of LC-PUFAs in improving behavioural, cognitive and physiological variables in children and adolescents and whether this intervention is efficacious.

## Competing interests

The authors declare that they have no competing interest.

## Authors’ contributions

CS, JS, AS, MK and JK conceived the study and developed the protocol. DC and RS are responsible for EEG protocol, analysis and interpretation. CS, AS and JK are responsible for behavioural data analysis and interpretation. CS is senior investigator and responsible for the overall trial. MK and JK are responsible for data collection. MK was Clinical Trials Coordinator for this study. All authors read and approved the final manuscript.

## References

[B1] Biederman J Attention-deficit/hyperactivity disorder: a selective overview Biol Psychiatry 2005 57 11 1215 1220 10.1016/j.biopsych.2004.10.020 15949990

[B2] American Psychiatric Association Diagnostic and statistical manual of mental disorders DSM-IV-TR 2000 4 Washington: American Psychiatric Publishing

[B3] Wolraich ML Wibbelsman CJ Brown TE Attention-deficit/hyperactivity disorder among adolescents: a review of the diagnosis, treatment, and clinical implications Pediatrics 2005 115 6 1734 1746 10.1542/peds.2004-1959 15930238

[B4] Curtis LT Patel K Nutritional and environmental approaches to preventing and treating autism and attention deficit hyperactivity disorder (ADHD): a review J Alter Complem Med 2008 14 1 79 85 10.1089/acm.2007.0610 18199019

[B5] Millichap JG Etiologic classification of attention-deficit/hyperactivity disorder Pediatrics 2008 121 2 358 365 10.1542/peds.2007-1332 18245408

[B6] Richardson AJ The importance of omega-3 fatty acids for behaviour, cognition and mood Scandinavian J Nutri Narings skning 2003 47 2 92 98 10.1080/11026480310007944

[B7] Di Michele F Prichep L John ER Chabot RJ The neurophysiology of attention-deficit/hyperactivity disorder Int J Psychophysiol 2005 58 1 81 93 10.1016/j.ijpsycho.2005.03.011 15979751

[B8] Chabot RJ Di Michele F Prichep L John ER The clinical role of computerized EEG in the evaluation and treatment of learning and attention disorders in children and adolescents J Neuropsychiatry Clin Neurosci 2001 13 2 171 186 10.1176/appi.neuropsych.13.2.171 11449024

[B9] Dvorakova M Jezova D Blazicek P Urinary catecholamines in children with attention deficit hyperactivity disorder (ADHD): modulation by a polyphenolic extract from pine bark (pycnogenol) Nutr Neurosci Jun-Aug 2007 10 3–4 151 157 10.1080/09513590701565443 18019397

[B10] Chabot RJ Serfontein G Quantitative electroencephalographic profiles of children with attention deficit disorder Biol Psychiatry 1996 40 10 951 963 10.1016/0006-3223(95)00576-5 8915554

[B11] Clarke AR Barry RJ McCarthy R Selikowitz M Electroencephalogram differences in two subtypes of attention-deficit/hyperactivity disorder Psychophysiology 2001 38 2 212 221 10.1111/1469-8986.3820212 11347867

[B12] Lazzaro I Gordon E Whitmont S Quantified EEG activity in adolescent attention deficit hyperactivity disorder Clin EEG Electroencephalogr 1998 29 1 37 42 10.1177/155005949802900111 9472424

[B13] Richardson AJ Clinical trials of fatty acid treatment in ADHD, dyslexia, dyspraxia and the autistic spectrum Prostaglandins Leukotri Essential Fatty Acids 2004 70 4 383 390 10.1016/j.plefa.2003.12.020 15041031

[B14] Ward PE Potential diagnostic aids for abnormal fatty acid metabolism in a range of neurodevelopmental disorders Prostaglandins Leukot Essent Fatty Acids 2000 63 1–2 65 68 10970715 10.1054/plef.2000.0193

[B15] Sinn N Bryan J Effect of supplementation with polyunsaturated fatty acids and micronutrients on learning and behavior problems associated with child ADHD J Dev Behav Pediatr 2007 28 2 82 91 10.1097/01.DBP.0000267558.88457.a5 17435458

[B16] Voigt RG Llorente AM Jensen CL Fraley JK Berretta MC Heird WC A randomized, double-blind, placebo-controlled trial of docosahexaenoic acid supplementation in children with attention-deficit/hyperactivity disorder J Pediatr 2001 139 2 189 196 10.1067/mpd.2001.116050 11487742

[B17] Sarris J Kean J Schweitzer I Lake J Complementary medicines (herbal and nutritional products) in the treatment of attention deficit hyperactivity disorder (ADHD): a systematic review of the evidence Complem Therap Med 2011 19 4 216 227 10.1016/j.ctim.2011.06.007 21827936

[B18] Cimera R Making ADHD a gift: teaching Superman how to fly 2002 Vol 16 Lanham: Scarecrow Press, Inc

[B19] El-Sayed E Larsson JO Persson HE Santosh PJ Rydelius PA “Maturational lag” hypothesis of attention deficit hyperactivity disorder: an update Acta Paediatrica Int J Paediat 2003 92 7 776 784 10.1080/08035250310002777 12892153

[B20] Biederman J Faraone SV Attention-deficit hyperactivity disorder Lancet 2005 366 9481 237 248 10.1016/S0140-6736(05)66915-2 16023516

[B21] Schmeichel BE Zemlan FP Berridge CW A selective dopamine reuptake inhibitor improves prefrontal cortex-dependent cognitive function: potential relevance to attention deficit hyperactivity disorder Neuropharmacology 2013 64 0 321 328 22796428 10.1016/j.neuropharm.2012.07.005 PMC3445755

[B22] Wallis D Russell HF Muenke M Review: genetics of attention deficit/hyperactivity disorder J Pediatr Psychol 2008 33 10 1085 1099 10.1093/jpepsy/jsn049 18522996

[B23] Swanson JM Kinsbourne M Nigg J Etiologic subtypes of attention-deficit/hyperactivity disorder: brain imaging, molecular genetic and environmental factors and the dopamine hypothesis Neuropsychol Rev 2007 17 1 39 59 10.1007/s11065-007-9019-9 17318414

[B24] McCann D Barrett A Cooper A Food additives and hyperactive behaviour in 3-year-old and 8/9-year-old children in the community: a randomised, double-blinded, placebo-controlled trial Lancet 2007 370 9598 1560 1567 10.1016/S0140-6736(07)61306-3 17825405

[B25] Spencer RC Klein RM Berridge CW Psychostimulants act within the prefrontal cortex to improve cognitive function Biol Psychiatry 2012 72 3 221 227 10.1016/j.biopsych.2011.12.002 22209638 PMC3319517

[B26] Simonoff E Taylor E Baird G Randomized controlled double-blind trial of optimal dose methylphenidate in children and adolescents with severe attention deficit hyperactivity disorder and intellectual disability J Child Psychol Psychiatry 2013 54 5 527 535 10.1111/j.1469-7610.2012.02569.x 22676856

[B27] Schachter HM Pham B King J Langford S Moher D How efficacious and safe is short-acting methylphenidate for the treatment of attention-deficit disorder in children and adolescents? A meta-analy Canadian Med Assoc J 2001 165 11 1475 1488 PMC81663 11762571

[B28] Sonuga-Barke EJS Coghill D Wigal T Debacker M Swanson J Adverse reactions to methylphenidate treatment for attention-deficit/hyperactivity disorder: structure and associations with clinical characteristics and symptom control J Child Adol Psychopharmacol 2009 19 6 683 690 10.1089/cap.2009.0024 20035586

[B29] Kemper KJ Vohra S The use of complementary and alternative medicine in pediatrics Pediatrics 2008 122 6 1374 1386 10.1542/peds.2008-2173 19047261

[B30] Rucklidge JJ Johnstone J Kaplan BJ Nutrient supplementation approaches in the treatment of ADHD Expert Rev Neurother 2009 9 4 461 476 10.1586/ern.09.7 19344299

[B31] Karpouzis F Bonello R Pollard H Chiropractic care for paediatric and adolescent attention-deficit/hyperactivity disorder: a systematic review 2010 1746 1340 10.1186/1746-1340-18-13 PMC2891800 20525195

[B32] Dyerberg J Linolenate-derived polyunsaturated fatty acids and prevention of atherosclerosis Nutr Rev 1986 44 4 125 134 2872640 10.1111/j.1753-4887.1986.tb07603.x

[B33] Burgess JR Stevens L Zhang W Peck L Long-chain polyunsaturated fatty acids in children with attention- deficit hyperactivity disorder Am J Clin Nutr 2000 71 1 SUPPL 327S 330S 10617991 10.1093/ajcn/71.1.327S

[B34] Kidd PM Omega-3 DHA and EPA for cognition, behavior, and mood: clinical findings and structural-functional synergies with cell membrane phospholipids Altern Med Rev 2007 12 3 207 227 18072818

[B35] Freeman MP Hibbeln JR Wisner KL Omega-3 fatty acids: evidence basis for treatment and future research in psychiatry J Clin Psychiatry 2006 67 12 1954 1967 10.4088/JCP.v67n1217 17194275

[B36] Fontani G Corradesschi F Felici A European journal of clinical investigation Eur J Clin Investig 2005 35 11 691 699 2005 10.1111/j.1365-2362.2005.01570.x 16269019

[B37] Hickman RW Menzel W *Perna canaliculus* (Gmelin) in New Zealand Estuarine and marine bivalve mollusk culture 1991 Boca Raton, FL: CRC Press 325 334

[B38] Croft J Relief from arthritis: a safe and effective treatment from the ocean 1980 Wellingbororough, Northampton, UK: Thorsons Publishers Ltd

[B39] Sinclair AJ Murphy KJ Li D Marine lipids: overview “news insights and lipid composition of Lyprinol” Allerg Immunol (Paris) 2000 32 7 261 271 11094639

[B40] Emelyanov A Fedoseev G Krasnoschekova O Abulimity A Trendeleva T Barnes PJ Treatment of asthma with lipid extract of New Zealand green-lipped mussel: a randomised clinical trial Eur Respir J 2002 20 3 596 600 10.1183/09031936.02.02632001 12358334

[B41] Whitehouse MW Macrides TA Kalafatis N Betts WH Haynes DR Broadbent J Anti-inflammatory activity of a lipid fraction (lyprinol) from the NZ green-lipped mussel Inflammopharmacology 1997 5 3 237 246 10.1007/s10787-997-0002-0 17638133

[B42] Cobb CS Ernst E Systematic review of a marine nutriceutical supplement in clinical trials for arthritis: the effectiveness of the New Zealand green-lipped mussel perna canaliculus Clin Rheumatol 2006 25 3 275 284 10.1007/s10067-005-0001-8 16220229

[B43] Walker-Bone K ‘Natural remedies’ in the treatment of osteoarthritis Drugs Aging 2003 20 7 517 526 10.2165/00002512-200320070-00004 12749749

[B44] Morelli V Naquin C Weaver V Alternative therapies for traditional disease states: osteoarthritis Am Fam Physician 2003 67 2 339 344 12562155

[B45] Kosuge T Tsuji K Ishida H Yamaguchi T Isolation of an antihistaminic substance from green-lipped mussel (Perna canaliculus) Chemical Pharmacology Bulletin 1986 34 4825 4828 10.1248/cpb.34.4825 3829196

[B46] Whitehouse MW Macrides TA Kalafatis N Anti-inflammatory activity of a lipid fraction (Lyprinol) from the NZ Green-lipped mussel Infammopharmacol 1997 5 237 246 1997 10.1007/s10787-997-0002-0 17638133

[B47] Macrides T Kalafatis N Lipid extract having anti-inflammatory activity 2002 http://www.ipaustralia.gov.au/. P. I. Limited. Australia. 35/56 (2006.01)

[B48] Lau CS Chiu PKY Chu EMY Treatment of knee osteoarthritis with Lyprinol®, lipid extract of the green-lipped mussel - A double-blind placebo-controlled study Prog Nutr 2004 6 1 17 31

[B49] Brien S Prescott P Coghlan B Systematic review of the nutritional supplement perna canaliculus (green-lipped mussel) in the treatment of osteoarthritis Quarter J Med 2008 101 167 179 2008 10.1093/qjmed/hcm108 18222988

[B50] Whitehouse MW Roberts MS Brooks PM Over the counter (OTC) oral remedies for arthritis and rheumatism: how effective are they? Inflammopharmacology 1999 7 2 89 105 10.1007/BF02918382 18597151

[B51] Conners CK A teacher rating scale for use in drug studies with children Am J Psychiatry 1969 126 6 884 888 4900822 10.1176/ajp.126.6.884

[B52] Conners CK Symptom patterns in hyperkinetic, neurotic, and normal children Child Development 1970 41 3 667 682 10.2307/1127215

[B53] Loo SK Barkley RA Clinical utility of EEG in attention deficit hyperactivity disorder Appl Neuropsychol 2005 12 2 64 76 10.1207/s15324826an1202_2 16083395

[B54] Llorente AM Voigt R Jensen CL Fraley JK Heird WC Rennie KM The test of variables of attention (TOVA): internal consistency (Q 1 vs. Q2 and Q3 vs. Q4) in children with attention deficit/hyperactivity disorder (ADHD) Child Neuropsychol 2008 14 4 314 322 10.1080/09297040701563578 17917866

[B55] Terry PC Lane AM Lane HJ Keohane L Development and validation of a mood measure for adolescents J Sports Sci 1999 17 11 861 872 1999/01/01 10.1080/026404199365425 10585166

[B56] Conners CK Sitarenios G Parker JDA Epstein JN The revised Conners’ parent rating scale (CPRS-R): factor structure, reliability, and criterion validity J Abnor Child Psychol 1998 26 4 257 268 10.1023/A:1022602400621 9700518

[B57] Kennedy DO Dodd FL Robertson BC Monoterpenoid extract of sage (Salvia lavandulaefolia) with cholinesterase inhibiting properties improves cognitive performance and mood in healthy adults J Psychopharmacol 2011 25 8 1088 1100 10.1177/0269881110385594 20937617

[B58] Kennedy DO Haskell CF Vitamins and cognition: what is the evidence? Drugs 2011 71 15 1957 1971 10.2165/11594130-000000000-00000 21985165

[B59] Kennedy DO Haskell CF Robertson B Improved cognitive performance and mental fatigue following a multi-vitamin and mineral supplement with added guarana (Paullinia cupana) Appetite 2008 50 506 513 2008 10.1016/j.appet.2007.10.007 18077056

[B60] Reay JL Kennedy DO Scholey AB The glycaemic effects of single doses of Panax ginseng in young healthy volunteers Br J Nutr 2006 96 639 642 2006 17010221

[B61] Reay JL Scholey AB Kennedy DO Panax ginseng (G115) improves aspects of working memory performance and subjective ratings of calmness in healthy young adults Hum Psychopharmacol 2010 25 6 462 471 10.1002/hup.1138 20737519

[B62] Scholey AB Harper S Kennedy DO Cognitive demand and blood gllucose Physiol Behav 2001 73 4 585 592 2001 10.1016/S0031-9384(01)00476-0 11495663

[B63] Scholey AB Kennedy DO Cognitive and physiological effects of an “energy drink”: an evaluation of the whole drink and of glucose, caffeine and herbal flavouring fractions Psychopharmacology 2004 176 3–4 320 330 2004 15549275 10.1007/s00213-004-1935-2

[B64] McNair DM Lorr M Droppleman LF Revised manual for the profile of mood states 1992 San Diego, CA: Services EaIT

[B65] Terry PC Lane AM Fogarty GJ Construct validity of the profile of mood states - adolescents for use with adults Psychol Sport Exer 2003 4 2 125 139 10.1016/S1469-0292(01)00035-8

[B66] Barry RJ Clarke AR Johnstone SJ Oades RD Electrophysiology in attention-deficit/hyperactivity disorder Int J Psychophysiol 2005 58 1 1 3 10.1016/j.ijpsycho.2005.03.003 15950305

[B67] Barry RJ Clarke AR McCarthy R Selikowitz M Brown CR Event-related potentials in children with attentiondeficit/hyperactivity disorder and excess beta activity in the EEG Acta Neuropsychologica 2009 7 4 249 263

[B68] Clarke AR Barry RJ Dupuy FE Behavioural differences between EEG-defined subgroups of children with attention-deficit/hyperactivity disorder Clin Neurophysiol 2011 122 7 1333 1341 10.1016/j.clinph.2010.12.038 21247797

[B69] Clarke AR Barry RJ Dupuy FE McCarthy R Selikowitz M Heaven PCL Childhood EEG as a predictor of adult attention-deficit/hyperactivity disorder Clin Neurophysiol 2011 122 1 73 80 10.1016/j.clinph.2010.05.032 20598939

[B70] Clarke AR Barry RJ McCarthy R Selikowitz M Age and sex effects in the EEG: Differences in two subtypes of attention-deficit/hyperactivity disorder Clin Neurophysiol 2001 112 5 815 826 10.1016/S1388-2457(01)00487-4 11336897

[B71] Clarke AR Barry RJ McCarthy R Selikowitz M Clarke DC Croft RJ EEG activity in girls with attention-deficit/hyperactivity disorder Clin Neurophysiol 2003 114 2 319 328 10.1016/S1388-2457(02)00364-4 12559240

[B72] Regan D Human brain electrophysiology: evoked potentials and evoked magnetic fields in science and medicine 1989 East Norwalk, Connecticut 06855: Appleton & Lange Publishing Company

[B73] Silberstein RB Ciorciari J Pipingas A Steady-state visually evoked potential topography during the wisconsin card sorting test Electroencephalogr Clin Neurophysiol 1995 96 1 24 35 10.1016/0013-4694(94)00189-R 7530186

