# Correction: A randomized controlled trial investigating the effects of PCSO-524®, a patented oil extract of the New Zealand Green Lipped Mussel (*Perna canaliculus*), on the behaviour, mood, cognition and neurophysiology of children and adolescents (aged 6–14 years) experiencing clinical and sub-clinical levels of hyperactivity and inattention: study protocol ACTRN12610000978066

**DOI:** 10.1186/1475-2891-12-113

**Published:** 2013-08-08

**Authors:** James D Kean, David Camfield, Jerome Sarris, Marni Kras, Richard Silberstein, Andrew Scholey, Con Stough

**Affiliations:** 1 Centre for Human Psychopharmacology, Swinburne University of Technology, PO Box 218 Hawthorn, Melbourne, Victoria 3122, Australia; 2 Department of Psychiatry, University of Melbourne, Melbourne, Australia

## 

The authors have identified an error in this published article [1].

On page 4, under “Treatments” the milligrams listed have been calculated incorrectly. The sentence:

“The Lyprinol capsules contain 18% EPA (46.8 mg) and 14% DHA (36.4 mg) per capsule.”

Should read:

“The Lyprinol capsules contain approximately 14% EPA (7.3 mg) and 11% DHA (5.5 mg) per capsule.”

We apologize for this error.
